# High blood pressure and overweight in children with Legg-Calvé-Perthes disease: a nationwide population-based cohort study

**DOI:** 10.1186/s12891-020-03889-9

**Published:** 2021-01-06

**Authors:** Gabriella B. Mörlin, Yasmin D. Hailer

**Affiliations:** Section of Orthopaedics, Department of Surgical Sciences, Section of Paediatric Orthopaedic Surgery, Uppsala University, Uppsala, Sweden

**Keywords:** Legg-Calvé-Perthes disease, Hypertension, BMI, Register, Overweight

## Abstract

**Purpose:**

Legg-Calvé-Perthes disease (LCPD) and its association with cardiovascular diseases, obesity and hypertension has been consistently observed but remains cloudy. This study aimed to investigate the presence of hypertension and overweight/obesity at diagnosis of LCPD and at a 2-year follow-up and its association with age, sex and lateral pillar classification.

**Method:**

We compared blood pressure (BP) (*n* = 93) and body mass index (BMI) (*n* = 125) in patients registered in the Perthes’ register - a part of the Swedish pediatric orthopedic quality register (SPOQ) - with normative data for children with the same age and sex.

**Results:**

In children with LCPD 19% had high BP. At the 2-year follow-up, 13% had high BP. For children with LCPD, 30% were either overweight or obese. At the 2-year follow-up, 32% were either overweight or obese. Paired analysis showed stable BMI z-score between these 2 measurements. The sample size of this study was too small to analyze possible associations of high BP or BMI with age, sex and lateral pillar classification.

**Conclusions:**

The prevalence of hypertension was higher in children with LCPD compared to general pediatric normative data. The same pattern was seen for overweight/obesity. Further studies are needed to investigate whether BP and obesity are catalyzing factors in the etiology of LCPD.

## Introduction

The etiology of Legg-Calvé-Perthes disease (LCPD) remains cloudy but a vascular component in the development of this condition, possibly in combination with mechanical stress, has been suggested [1] amongst other numerous hypotheses [2]. The disease typically occurs in children aged 5–8 years [3, 4] and is 3–4 times more common in boys [4, 5]. The incidence of LCPD ranges from 0.5 to 100,000 in South Africa [6] to 21 per 100,000 in the Liverpool area [7]. Geographical variations are described with increasing incidences with increasing latitudes [8].

In Sweden, the incidence of LCPD has been estimated at approximately nine per 100,000 [9]. Perry et al. found in a sex- and age-matched case-control study alterations in arterial blood flow after ischemic stimulus [10]. Specifically, the authors observed a significant structural alteration in the vasculature and reduction in blood flow and velocity in children with LCPD. In addition, hypertension has been identified as a potential catalyzing factor in LCPD [11] and associations with cardiovascular diseases, including hypertension have been described in adult patients with a history of LCPD [12]. Blood pressure (BP) measurement is generally not an integral part of the children’s surveillance program in Sweden. Thus, it is still unclear whether hypertension is prevalent at the time LCPD is diagnosed and therefore a potential promoter of the disease or whether it occurs first in adulthood.

Another etiological pathway is body composition in patients with LCPD. However, there are spurious and conflicting findings regarding overweight and obesity. In some older studies children with LCPD have been described with low birth weight, skeletal retardation [13] and short body length [14]. In contrast, more recent studies have reported normal body habitus [15] or a rather high prevalence of overweight and obesity [16], including a higher risk of developing obesity later in life with a mean delay of 10 years [17]. Furthermore, childhood obesity is a risk factor for childhood hypertension [18] and is associated with metabolic syndrome, cardiovascular diseases, type 2 diabetes and its associated complications [19].

The overall aims of this study were to show (i) whether children with LCPD have high BP at the time of LCPD diagnosis and at 2-years follow-up and if BP is associated with age, sex or Lateral Pillar classification. In addition, (ii) whether children with LCPD are overweight or obese at time of LCPD diagnosis and at 2-years follow-up and if overweight or obesity is associated with age, sex or Lateral Pillar classification.

## Methods

This nationwide population-based cohort study was performed using the LCPD register, which is a part of the Swedish National Quality Register for Pediatric Orthopedics (SPOQ). The SPOQ contains epidemiological data for children diagnosed with LCPD. All patients registered from 1 April 2015, when the register was initiated, until 31 December 2019 were included in the study. Inclusion criteria were patients with new diagnosed LCPD (ICD M91.1), with a Swedish personal identity number and who were treated in Sweden since diagnosis. The diagnosis must have been completed between the ages $\ge$2 and <13 years and confirmed radiologically between 1 January 2015 and 31 December 2019. The patients were registered on the following occasions: at diagnosis, time of possible primary surgery, times of possible reoperation, 2 years after diagnosis and at the ages of 10 years. Patients with secondary avascular necrosis of the femoral head are not included in the register. While length, weight and BP were optional to register in SPOQ between 2015 and 2017, these variables became mandatory to register in 2018.

### Characteristics of the study population and reference group

The initial database contained 199 hips of 192 patients, 42 girls and 150 boys. In total 23 patients were diagnosed with bilateral LCPD, of these, 7 had both hips registered in SPOQ while the others had the contralateral hip diagnosed before the register was established. Both sides were affected to the same extent. The mean age at LCPD diagnosis was 5.9 (range 2.0-12.2) in boys and 6.4 (range 2.2–11.7) in girls. 56 patients received containment surgery. All characteristics of the study population are shown in Table 1.

**Table 1:** Characteristic of the study population

n	192
Age at diagnosis (mean (SD))	6.0 (2.2)
BMI at diagnosis (mean (SD)), *n* = 125	16.9 (2.7)
BMI_z-score at diagnosis (mean (SD)), *n* = 125	0.4 (1.3)
Systolic Blood Pressure at diagnosis (mean (SD)), *n* = 93	102.9 (12.4)
Systolic Blood Pressure_z-score at diagnosis (mean (SD)), *n* = 93	1.1 (5.5)
Diastolic Blood Pressure at diagnosis (mean (SD)), *n* = 93	63.3 (9.6)
Diastolic Blood Pressure z-score at diagnosis (mean (SD)), *n* = 93	0.6 (0.8)
Weight at diagnosis (mean (SD))	23.1 (7.2)
Weight z.score at diagnosis (mean (SD))	0.4 (1.2)
Height at diagnosis (mean (SD))	116.1 (14.0)
Height_z-score at diagnosis (mean (SD))	0.2 (1.2)
LCPD Side (%)	
Right	95 (49.5)
Left	97 (50.5)
Lateral pillar at diagnosis (%)	
A	3 (1.6)
B	65 (33.9)
B/C	23 (12.0)
C	42 (21.9)
too early	59 (30.7)
Surgery (%)	56 (29.2)

For a sensitivity analysis, we only included patients with unilateral affection, 39 girls and 137 boys. We followed the study population from 15 to 2015 when the SPOQ-register was established until 31 December 2019. Of the 192 patients in SPOQ, 107 had been in the register for ≥ 2 years. Of these, 77 patients had a 2-year follow-up registered in SPOQ.

According to the growth charts of the Centers for Disease Control and Prevention (CDC), overweight in children is defined as a BMI ≥ 85th to < 95th percentile and obesity as a BMI ≥ 95th percentile [20]. The approach to defining hypertension in children has been to use percentile rank and define BP elevation as BP ≥ 95th percentile [21]. The medical definition of normotensive in children is < 90th percentile and pre-hypertensive ≥ 90th to < 95th percentile. The calculation of z-scores and exact percentiles is based on the recommended growth charts by the CDC and for children and adolescents 2–19 years of age. The CDC growth charts are the improved version of the US National Center for Health Statistics. The National Health and Nutrition Examination Surveys (NHANES), which is a series of cross-sectional, national, stratified, probability surveys, of the population in the US, have updated the reference data together with statistical procedures [20]. BP z-scores and percentiles, based on percentiles of height, age and sex, were calculated and interpreted in accordance with the Fourth Report on the Diagnosis, Evaluation and Treatment of High Blood Pressure in Children and Adolescents. These clinical practice guidelines have been established by combining and reexamining the US national database on normative BP levels throughout childhood with data from the 1999–2000 NHANES. All measurements are based on the auscultation process, which is the recommended method of obtaining BP. We compared the patients with missing BP data and BMI data and did not find major differences to patients with registered data (Tables 2 and 3). Based on previous prevalence studies on BP and overweight/obesity (P) in children [22–24], the estimated sample size (n) (calculated with *n* = Z2P(1 − P)/d2, where d is precision) is 71 patients for hypertension, 208 for overweight and 45 for obesity.

**Table 2:** Comparison of study populations with missing blood pressure registration and registered blood pressure

	Missing values	Registered BP	*p*-value
n	99	93	
Age (mean (SD))	5.7 (2.3)	6.4 (2.1)	0.03
Sex = Female (%)	23 (23.2)	19 (20.4)	0.77
Side = left (%)	47 (47.5)	50 (53.8)	0.47
Lateral Pillar (%)			0.30
A	1 (1.0)	2 (2.2)	
B	35 (35.4)	30 (32.3)	
B/C	16 (16.2)	7 (7.5)	
C	21 (21.2)	21 (22.6)	
too early	26 (26.3)	33 (35.5)

**Table 3:** Comparison of study populations with missing registration for BMI versus registered BMI

	Missing values	Registered BMI	*p*-value
n	67	125	
Age (mean (SD))	5.9 (2.4)	6.1 (2.2)	0.5
Sex = Female (%)	13 (19.4)	29 (23.2)	0.7
Side = left (%)	32 (47.8)	65 (52.0)	0.7
Lateral Pillar (%)			0.6
A	1 (1.5)	2 (1.6)	
B	24 (35.8)	41 (32.8)	
B/C	11 (16.4)	12 (9.6)	
C	12 (17.9)	30 (24.0)	
too early	19 (28.4)	40 (32.0)	

Continuous data were described using means, medians, and ranges. Categorical data were cross tabulated and proportions were investigated using the chi-square test. Paired analyses were performed with the Wilcoxon rank-sum test. Differences were considered significant if the p-value was ≤ 0.05. Logistic regression analyses were performed to estimate the Odd’s ratio (OR) with 95% Confidence interval (CI) for hypertension or overweight/obesity in association to age, sex and Lateral Pilar Classification at diagnosis and at fragmentation stage. All statistical analyses were performed using R statistic software (Version 3.5.1; R Foundation for Statistical Computing, Vienna, Austria), including the “powerAnalysis”, “rms”, “magrittr”, “ggplot2”, “ggpubr”, “tidyverse” and “Gmisc” packages.

## Results

Of all patients registered in SPOQ, BP was registered in 48.4% (n = 93) of the children with LCPD. Of these, 19% (n = 18) had either high systolic (n = 7) or diastolic BP (n = 6) or both (5 children) at or beyond the 95th percentile. Logistic regression analyzes revealed and OR of 1.8 between high BP and female sex, an OR of 1.9 between high BP and overweight but the 95% CI were broad and did not reach significant level. The ORs between BP and age or lateral pillar classification at diagnosis were around 1.0 (Table 4).

**Table 4:** Logistic regression analyses for hypertension (> 95 percentile) and overweight/obesity (> 85 percentile) in association to sex, age, Lateral Pilar Classification at fragmentation stage and having had surgery

	OR	CI: 2.5%	CI: 97.5%	*p*-value
**High BP (> 95 percentile)**				
Sex (female)	1.8	0.5	5.9	0.4
Age	0.9	0.7	1.2	0.5
Lateral Pillar	0.9	0.6	1.4	0.7
Surgery	1.3	0.4	4.8	0.7
Overweight/Obesity	1.9	0.6	5.8	0.3
**High BMI (> 85 percentile**				
Sex (female)	1.2	0.4	3.6	0.8
Age	1.0	0.7	1.2	0.8
Lateral Pillar	1.0	0.7	1.4	0.8
Surgery	0.7	0.2	2.0	0.5

**Fig. 1:**
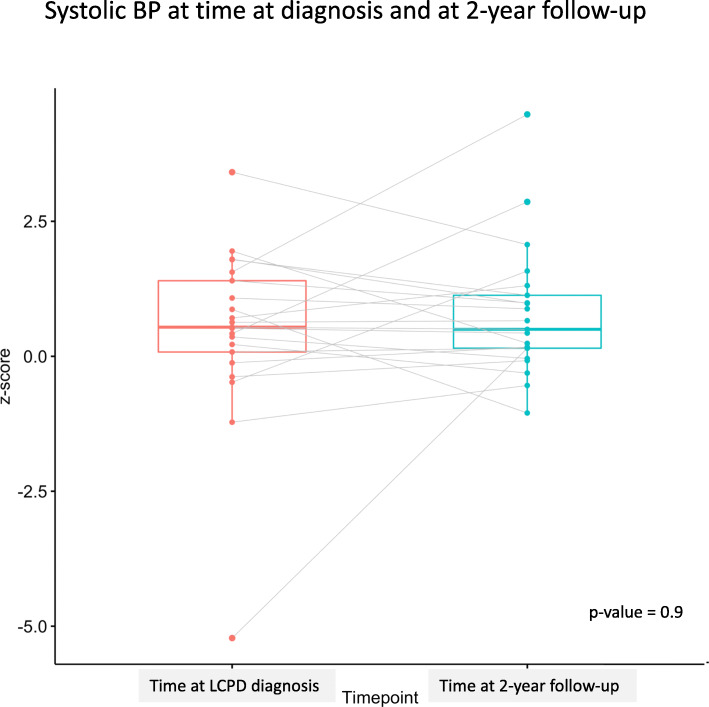
Systolic BP at time at diagnosis and at 2-year follow-up

**Fig. 2:**
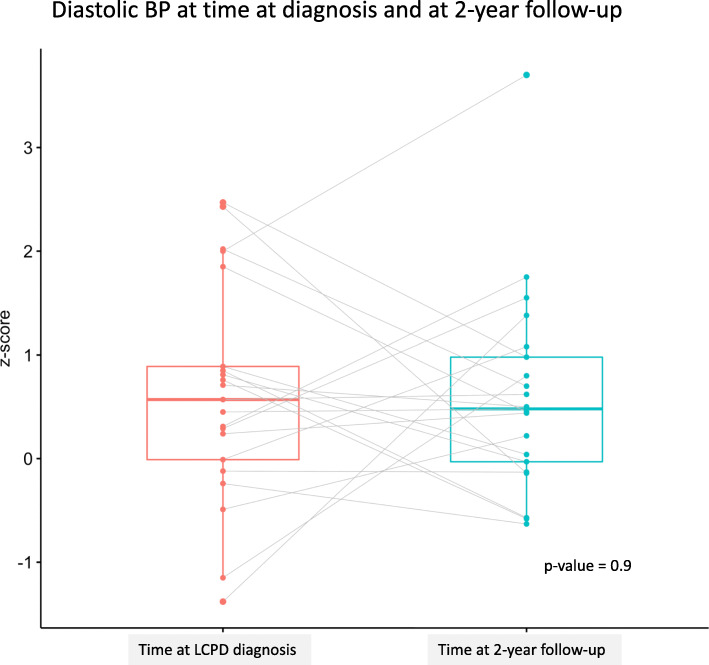
Diastolic BP at time at diagnosis and at 22-year follow-up

Of the 77 patients who had the 2-year follow-up after diagnosis registered in SPOQ, 45 children (58%) had their BP registered. Of these, 13% (*n* = 6) had high BP (≥ 95th percentile). A paired analysis showed no changes in the z-scores for BP levels when comparing the scores at the time of diagnosis with those at the 2-year follow-up (Figs. 1 and 2).

BMI was registered in 65.1% (*n* = 125) of the children with LCPD. Of these, 30% were either overweight (21 children) or obese (17 children). In logistic regression analysis the ORs between high BMI and sex, age at diagnosis or lateral pillar classification or treatment were all around 1.0 (Table 4).

**Fig. 3:**
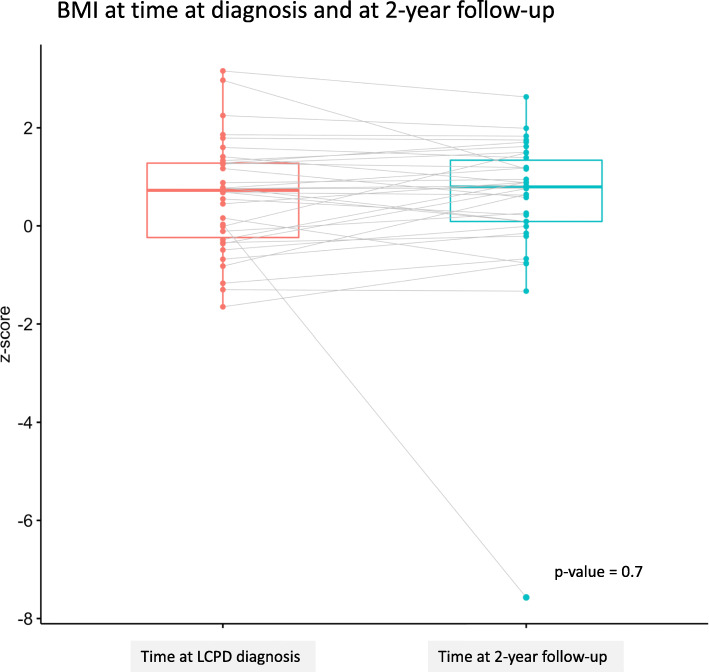
BMI at time at diagnosis and at 2-year follow-up

At the 2-year follow-up, BMI was registered in 50 children (65%). Of these 50 children, 8 were overweight and 8 obese. A paired analysis showed no significant changes in BMI-z-scores when comparing measurements at the time of diagnosis with those obtained at the 2-year follow-up (Fig. 3). Stratified analysis for therapy (non-surgical versus surgical) did not change the results.

A sensitivity analysis, in which patients diagnosed with bilateral LCPD were excluded, did not meaningfully change the results (data not shown).

## Discussion

The estimated prevalence of high BP in this study was 19% at time at diagnosis of LCPD and 13% at 2-years follow-up. In contrast to the findings from Kit et al. who described a higher prevalence of high BP in older but otherwise healthy children [25], we found no association to age which is most likely because of our small sample size. Studies in the US estimate the prevalence of hypertension in a pediatric population to range from 2–5% [22, 23]. To our knowledge, no reliable statistics are available reporting the prevalence of hypertension in Swedish children. We know that hypertension can induce LCPD in rats [11] and that children with LCPD have abnormal vessels and blood flow [10]. In addition, patients with a history of LCPD had a 2.2-fold higher risk of hypertension [12] but hypertension was most often diagnosed in adulthood. However, because it is mostly asymptomatic, hypertension in children is highly underdiagnosed, especially in those who are overweight [26]. We believe the present study is the first to measure hypertension at the time LCPD was diagnosed. Even though we do not have blood pressure measurements before the diagnosis of LCPD, the assumption that hypertension could be a catalyzing factor in the etiology of LCPD needs to be investigated further.

The prevalence of overweight and obesity in children with LCPD was 30% at diagnosis and 32% at the 2-year follow-up. The prevalence of obesity at LCPD diagnosis corresponds to the findings of Neal et al. [16]. In a Swedish study of almost 4600 school children from the ages 7–9 years the overall prevalence of overweight was estimated at 17% and obesity at 3% [24]. An increased risk of obesity in patients with LCPD has been demonstrated in a population-based cohort study in Sweden [17]. However, obesity in that cohort was mostly diagnosed after the diagnosis of LCPD. Physical inactivity as a result of hip pain has been discussed as a potential factor to account for overweight [16]. If this was the case we would expect an increase of BMI within the 2 years between diagnosis and follow-up of LCPD patients. However, in this study, no increase of BMI from the time at diagnosis of LCPD to the 2 years follow-up was seen which makes it unlikely that physical inactivity due to pain might be the sole cause for overweight and obesity. Children with LCPD are prone to express hyperactive behavior with an increased risk of attention deficit hyperactivity disorder (ADHD) [26, 27] which might be one explanation for a steady BMI. Additionally, a meta-analysis found a > 30% higher pooled prevalence of obesity in children with ADHD compared with those without ADHD [27]. Cortese et al. [28] discussed possible abnormal eating patterns as the underlying mechanism that could even apply to children with LCPD. Another pathway is the association between overweight and higher fat-derived hormone (e.g., levels of leptin in LPCD patients have been reported to be higher with the severity of the disease, [29]), which might explain the higher prevalence of overweight and obesity in LCPD patients.

Generally, BP is strongly associated with BMI and higher BP levels are associated with high BMI [26, 30, 31]. Children with a BMI beyond the 85th percentile had an OR of 1.9 of having high BP (≥ 95th percentile) with a broad confidence interval which did not reach significance level. Again, the number of patients in this study is too small to draw any conclusions about an association between BMI and BP in children with LCPD. Therefore, the findings need to be confirmed in a larger cohort.

### Limitations

We used data on Swedish children with LCPD from the SPOQ and compared these data to sex- and age-specific reference data from the US. We did so because the US reference data included z-scores that offer an analysis of extreme values, which was preferred. International differences might be apparent but Krmar et al. did not find any significant difference in BP between the American and Swedish datasets [32].

Another potential source of error might be the routines of measuring BP and weight. For BP, the data are based on one single measurement and not as recommended, i.e. taking the mean of three measurements. Weight was taken at the time of the orthopedic consultation visit. Different hospitals might not use the same equipment and also that measurements took place at different times of the day. The SPOQ does provide measurement instructions for their use; however, it remains uncertain whether the instructions are strictly followed.

Regrettably, measurements of BP, height and weight were only mandatory in the SPOQ in 2018, which resulted in many patients with missing data on these parameters before and after 2018. However, a subgroup analysis demonstrated that patients with missing data did not differ strongly in sex and age compared with patients with registered data (Tables 2 and 3) neither did the prevalence of BP (13%) and overweight/obesity (31%) in 2018 when these measurements were mandatory to register. Still, to detect associations between BP or BMI and other covariates such as sex or the severity of LCPD with and a power of 0.8 and an effect size of > 0.2 more patients are needed. On the other hand, this study is the first nationwide cohort study based on a quality register for LCPD with special focus on hypertension and overweight/obesity. The results of this hypothesis-generating study need to be confirmed in studies with a confirmatory design.

## Conclusions

Children patients with LCPD who were registered in the SPOQ's register for Perthes’ disease in 2015–2019 had higher prevalence of hypertension as compared with the estimated prevalence of hypertension among the general pediatric population. The assumption that hypertension might be a catalyzing factor in LCPD - as shown in experimental studies - is therefore possible but further studies are needed to investigate a potential relationship between hypertension, obesity and LCPD in children. As hypertension during childhood is strongly associated with cardiovascular diseases in adulthood, controls of BP levels through the children surveillance program in Sweden should be discussed.

We also found a higher prevalence of overweight and obesity than the estimated prevalence of overweight and obesity in the Swedish pediatric population at both diagnosis and the 2-year follow-up.

## Data Availability

The dataset analyzed in this study is not publicly available as the study was approved on the ground of ensuring the confidentiality of data of patients included in the study.

## Abbreviations

LCPD: Legg-Calvé-Perthesdisease
BP: bloodpressure
SPOQ: Swedishpediatric orthopedic quality register
BMI: Body mass index
CDC: Centersfor Disease Control and Prevention
NHANES: NationalHealth and Nutrition Examination Surveys
OD: Odd’sratio
CI: Confidenceinterval
ADHD: Attentiondeficit hyperactivity disorder

## References

[1] Wynne-Davies R, Gormley J (1978). The aetiology of Perthes’ disease. Genetic, epidemiological and growth factors in 310 Edinburgh and Glasgow patients. J Bone Joint Surg Br.

[2] Pavone V, Chisari E, Vescio A, Lizzio C, Sessa G, Testa G (2019). Aetiology of Legg-Calvé-Perthes disease: A systematic review. World J Orthop.

[3] Perry DC, Hall AJ. The epidemiology and etiology of Perthes disease. Orthop Clin North Am. 2011;42:279–83, v. 10.1016/j.ocl.2011.03.002 21742139

[4] Wiig O, Terjesen T, Svenningsen S, Lie SA (2006). The epidemiology and aetiology of Perthes’ disease in Norway. A nationwide study of 425 patients. J Bone Joint Surg Br.

[5] Guille JT, Lipton GE, Szoke G, Bowen JR, Harcke HT, Glutting JJ (1998). Legg-Calve-Perthes disease in girls. A comparison of the results with those seen in boys. J Bone Joint Surg Am.

[6] Purry NA (1982). The incidence of Perthes’ disease in three population groups in the Eastern Cape region of South Africa. J Bone Joint Surg Br.

[7] Hall AJ, Barker DJ, Dangerfield PH, Taylor JF (1983). Perthes’ disease of the hip in Liverpool. Br Med J (Clin Res Ed).

[8] Perry DC, Machin DMG, Pope D, Bruce CE, Dangerfield P, Platt MJ (2012). Racial and geographic factors in the incidence of Legg-Calvé-Perthes’ disease: a systematic review. Am J Epidemiol.

[9] Johansson T, Lindblad M, Bladh M, Josefsson A, Sydsjo G (2017). Incidence of Perthes’ disease in children born between 1973 and 1993. Acta Orthop.

[10] Perry DC, Green DJ, Bruce CE, Pope D, Dangerfield P, Platt MJ (2012). Abnormalities of vascular structure and function in children with Perthes disease. Pediatrics.

[11] Hirano T, Iwasaki K, Yamane Y (1988). Osteonecrosis of the femoral head of growing, spontaneously hypertensive rats. Acta Orthop Scand.

[12] Hailer YD, Montgomery SM, Ekbom A, Nilsson OS, Bahmanyar S (2010). Legg-Calve-Perthes disease and risks for cardiovascular diseases and blood diseases. Pediatrics.

[13] Lappin K, Kealey D, Cosgrove A, Graham K (2003). Does low birthweight predispose to Perthes’ disease? Perthes’ disease in twins. J Pediatr Orthop B.

[14] Burwell RG (1988). Perthes’ disease: growth and aetiology. Arch Dis Child.

[15] Kealey WD, Lappin KJ, Leslie H, Sheridan B, Cosgrove AP (2004). Endocrine profile and physical stature of children with Perthes disease. J Pediatr Orthop.

[16] Neal DC, Alford TH, Moualeu A, Jo CH, Herring JA, Kim HK (2016). Prevalence of Obesity in Patients With Legg-Calve-Perthes Disease. J Am Acad Orthop Surg.

[17] Hailer YD, Hailer NP (2018). Is Legg-Calvé-Perthes Disease a Local Manifestation of a Systemic Condition?. Clin Orthop Relat Res.

[18] Aguilar-Cordero MJ, Rodríguez-Blanque R, Leon-Ríos X, Expósito Ruiz M, García García I, Sánchez-López AM (2020). Influence of Physical Activity on Blood Pressure in Children With Overweight/Obesity: A Randomized Clinical Trial. Am J Hypertens.

[19] Kelsey MM, Zaepfel A, Bjornstad P, Nadeau KJ (2014). Age-related consequences of childhood obesity. Gerontology.

[20] Kuczmarski RJ, Ogden CL, Guo SS, Grummer-Strawn LM, Flegal KM, Mei Z, et al. 2000 CDC Growth Charts for the United States: methods and development. Vital Health Stat. 2002;11:1–190. 12043359

[21] The fourth report on the diagnosis, evaluation, and treatment of high blood pressure in children and adolescents. Pediatrics. 2004;114 2 Suppl 4th Report:555–76. 15286277

[22] Sorof JM, Lai D, Turner J, Poffenbarger T, Portman RJ (2004). Overweight, ethnicity, and the prevalence of hypertension in school-aged children. Pediatrics.

[23] Moore WE, Stephens A, Wilson T, Wilson W, Eichner JE (2006). Body mass index and blood pressure screening in a rural public school system: the Healthy Kids Project. Prev Chronic Dis.

[24] Sjöberg A, Moraeus L, Yngve A, Poortvliet E, Al-Ansari U, Lissner L (2011). Overweight and obesity in a representative sample of schoolchildren - exploring the urban-rural gradient in Sweden. Obes Rev.

[25] Kit BK, Kuklina E, Carroll MD, Ostchega Y, Freedman DS, Ogden CL (2015). Prevalence of and trends in dyslipidemia and blood pressure among US children and adolescents, 1999–2012. JAMA Pediatr.

[26] Hansen ML, Gunn PW, Kaelber DC (2007). Underdiagnosis of hypertension in children and adolescents. JAMA.

[27] Cortese S, Moreira-Maia CR, St Fleur D, Morcillo-Peñalver C, Rohde LA, Faraone SV (2016). Association Between ADHD and Obesity: A Systematic Review and Meta-Analysis. Am J Psychiatry.

[28] Cortese S, Tessari L (2017). Attention-Deficit/Hyperactivity Disorder (ADHD) and Obesity: Update 2016. Curr Psychiatry Rep.

[29] Lee JH, Zhou L, Kwon KS, Lee D, Park BH, Kim JR (2013). Role of leptin in Legg-Calve-Perthes disease. J Orthop Res.

[30] Wang M, Kelishadi R, Khadilkar A, Mi Hong Y, Nawarycz T, Krzywińska-Wiewiorowska M (2020). Body mass index percentiles and elevated blood pressure among children and adolescents. J Hum Hypertens.

[31] Lawlor DA, Najman JM, Sterne J, Williams GM, Ebrahim S, Davey Smith G (2004). Associations of parental, birth, and early life characteristics with systolic blood pressure at 5 years of age: findings from the Mater-University study of pregnancy and its outcomes. Circulation.

[32] Krmar RT, Holtbäck U, Bergh A, Svensson E, Wühl E (2015). Oscillometric casual blood pressure normative standards for Swedish children using ABPM to exclude casual hypertension. Am J Hypertens.

